# Is Chronotype Related to Eating Distractions Among Adolescents? The EHDLA Study

**DOI:** 10.3390/nu18172763

**Published:** 2026-08-24

**Authors:** Martina Jared Masache-Cruz, Fiorella Quiroz-Cárdenas, Camila Miño, Emily Cisneros-Vásquez, José Adrián Montenegro-Espinosa, Lucille Ridgell, Rodrigo Yáñez-Sepúlveda, Chih-Fu Wei, Miguel López-Moreno, José Francisco López-Gil

**Affiliations:** 1 School of Medicine, Universidad Espíritu Santo, Samborondón 0901952, Ecuador; martinamasache.09@gmail.com; 2 Vicerrectoría de Investigación y Postgrado, Universidad de Los Lagos, Osorno 5290000, Chile; fiorella.quiroz@yahoo.com; 3 Department of Health Research, Icen Cognis, 38370 La Matanza de Acentejo, Spain; 4 European Institute of Higher Studies, IEES, 4824-909 Fafe, Portugal; camilamino501@gmail.com; 5 Universidad Internacional para el Desarrollo (UNINDE), 06080 Badajoz, Spain; emily.sofy@hotmail.com; 6 Universidad Autónoma del Paraguay, Asunción 001010, Paraguay; josemontenegro733@gmail.com; 7 Faculty of Health Sciences, Universidad Autónoma de Chile, Temuco 4810101, Chile; lucilleridgell@gmail.com; 8 Faculty of Education and Social Sciences, Universidad Andrés Bello, Viña del Mar 2520000, Chile; rodrigo.yanez.s@unab.cl; 9 Taiwan Technical Mission to Tuvalu, Funafuti P.O. Box 130, Tuvalu; chihfuwei@gmail.com; 10 Department of Environmental and Occupational Medicine, National Taiwan University Hospital Yunlin Branch, Yunlin 640203, Taiwan; 11 Department of Environmental and Occupational Medicine, National Taiwan University College of Medicine and Hospital, Taipei 10051, Taiwan; 12 Diet, Planetary Health and Performance, Faculty of Health Sciences, Universidad Francisco de Vitoria, 28223 Madrid, Spain; miguel.lopez@ufv.es; 13 Faculty of Health Sciences, Universidad Tecnológica Atlántico Mediterráneo—UTAMED, 29590 Málaga, Spain

**Keywords:** mindful eating, circadian preference, chronobiology, teenagers, Spain

## Abstract

Background: Screen-based distractions during meals are increasingly common during adolescence and have been linked to less healthy eating behaviors. Chronotype, an individual’s circadian preference for morningness or eveningness, is associated with differences in alertness, self-regulation, and recreational screen use across the day, which may in turn influence susceptibility to distractions during meals. However, the relationship between chronotype and eating distraction behaviors remains largely unexplored. Therefore, this study examined the association between chronotype and eating distraction behaviors in Spanish adolescents. Methods: A cross-sectional study was carried out in 820 adolescents aged 12–17 years from the Eating Healthy and Daily Life Activities (EHDLA) project. Chronotype was assessed using the Morningness–Eveningness Scale for Children and classified as evening, intermediate, or morning type. Eating distractions were evaluated using a composite score that included eating lunch or dinner while watching television, using mobile devices or social networks during meals, and eating while standing. Associations were examined using ordinal regression models (proportional odds), with eveningness as the reference category; both unadjusted and adjusted models are reported, with adjustment for relevant sociodemographic and lifestyle covariates. Results: In adjusted ordinal regression models with eveningness as reference, morningness was related to lower odds of being in a higher eating distractions category (odds ratio [OR] = 0.53; 95% confidence interval [CI] 0.35–0.82; *p* = 0.004). Morningness was also associated with lower odds of a higher category of television viewing during meals (OR = 0.59; 95% CI 0.38–0.93; *p* = 0.024). No significant associations were identified for eating while using a phone or social networks or for eating while standing. Conclusions: Chronotype was independently associated with eating distraction behaviors in Spanish adolescents, with evening types showing a less favorable pattern. These findings suggest that circadian preference may be relevant when addressing mealtime behaviors during adolescence and could help inform future health promotion strategies.

## 1. Introduction

Distractions during eating, particularly screen-based activities such as television viewing or smartphone use, have become increasingly prevalent during adolescence [1,2]. Experimental studies have shown that cognitively demanding activities performed while eating can increase energy intake and reduce awareness of food consumption [3,4]. Furthermore, reduced attention to food has been related to poorer dietary quality, greater consumption of sugar-sweetened beverages, and greater likelihood of overweight and cardiometabolic disturbances [5]. In this context, eating distractions can be defined as any activity that competes for an individual’s attention during food consumption, such as watching television, standing while eating, or using mobile devices [6]. Interventions promoting attentive or mindful eating suggest that minimizing external stimuli during meals may improve dietary behavior and reduce subsequent energy intake. Beyond environmental influences, the timing of meals and individual differences in circadian preference may also be associated with variability in eating distraction behaviors [7]. It is important to note that eating behaviors are also regulated by biological mechanisms including appetite-regulating hormones such as leptin, adiponectin, and ghrelin, as well as individual differences in eating speed [8]. Environmental and behavioral factors such as screen use during meals should therefore be understood as one of several contributors to eating behavior, rather than a sole determinant [6].

Chronotype refers to an individual’s biological predisposition towards a specific circadian phase within the sleep–wake cycle, typically classified as morning or evening preference [9,10]. Circadian rhythms regulate multiple behavioral and physiological processes, including sleep quality, appetite regulation, and dietary timing [11]. Chronotype has also been associated with cardiovascular health. Circadian preference has been shown to influence the expression of clock-related genes that regulate metabolic processes. Dysregulation of these genes may disrupt internal circadian alignment and impair reward-related neural mechanisms, fostering unhealthy lifestyle behaviors such as irregular sleep patterns, poor dietary quality, and ultimately increasing cardiovascular risk [12]. Adolescence represents a particularly vulnerable period due to increased susceptibility to social jetlag, defined as the misalignment between social rhythms and biological schedules, which has been associated with metabolic disturbances and unhealthy lifestyle behaviors [13]. Evidence suggests that chronotype is associated with eating patterns, with evening-oriented individuals more likely to skip breakfast, consume energy-dense foods, and display less balanced dietary habits [9,14]. Consistent with this, studies in adolescent populations have reported poorer sleep habits, higher obesity prevalence, and lower quality of life among evening and intermediate chronotypes [15]. Given these behavioral differences, chronotype may also be related to contextual aspects of eating, including susceptibility to distractions during meals.

Despite extensive research linking chronotype to dietary behaviors and lifestyle patterns, little is known about whether circadian preference is associated with contextual eating behaviors such as susceptibility to distractions during meals. Research suggests that chronotype is associated with variations in alertness, cognitive performance, and attentional regulation across the day [16], which may be related to differences in how individuals engage with their environment during eating occasions. Furthermore, studies linking evening chronotype with attentional difficulties and later circadian rhythms provide indirect evidence that circadian preference could be linked to mealtime behaviors. Despite these indications, the relationship between chronotype and eating distractions remains largely unexplored, particularly in adolescent populations.

Several pathways may link chronotypes to eating distractions. Evening-oriented individuals tend to experience greater social jetlag and shorter sleep duration, both of which are associated with reduced executive functioning and impaired self-regulation, potentially increasing susceptibility to environmental distractions during meals [17]. Evening chronotype has also been consistently linked to greater recreational screen use, which may independently increase exposure to television or smartphone use during mealtimes [9,18]. For this reason, sleep duration and screen-related behaviors were included as covariates rather than as mediators in the present analysis. Understanding whether chronotype is associated with these contextual eating behaviors is particularly relevant during adolescence, a developmental stage characterized by increased screen exposure and irregular daily schedules. To date, no study has examined this relationship in youths. Therefore, the aim of this study was to investigate the association between chronotypes (independent variable) and eating distractions (dependent variables) in a sample of Spanish adolescents.

## 2. Materials and Methods

### 2.1. Participants and Study Design

The Eating Healthy and Daily Life Activities (EHDLA) project is a cross-sectional study involving Spanish adolescents aged 12–17 years recruited from high schools in *Valle de Ricote* (Region of Murcia) during the 2021–2022 academic year. Data collection was carried out during physical education classes. A complete description of the EHDLA study protocol was previously published [19].

Inclusion criteria were ages between 12 and 17 years, residence or school enrollment in the *Valle de Ricote*, and provision of written informed consent from parents/legal guardians. Participants with medical conditions or physical activity restrictions that could interfere with study procedures were excluded; neurodevelopmental and psychiatric diagnoses (e.g., attention-deficit/hyperactivity disorder (ADHD), autism spectrum disorder, depression, anxiety, eating disorders, or sleep disorders) were not systematically assessed or used as exclusion criteria. The initial sample comprised 1378 students. Participants with missing data on chronotype (*n* = 478; 34.7%), anthropometric data (*n* = 117; 8.5%), or other analytical covariates/outcomes were excluded, yielding a final analytical sample of 820 adolescents (40.5% of the initial sample excluded due to incomplete data). A comparison of included and excluded participants is provided in Table S5. The study was approved by the Ethics Committee of the Albacete University Hospital Complex and the Albacete Integrated Care Management (ID 2021-85), as well as Bioethics Committee of the University of Murcia (ID 2218/2018), and was conducted in accordance with the Helsinki Declaration.

### 2.2. Procedures

#### 2.2.1. Eating Distractions

In this study, eating distractions were defined as any activity that competes for an individual’s attention during food consumption, such as multitasking activities. The eating distraction score combined three behaviors: (a) using mobile devices during meals (e.g., phone calls, messaging, or social media use); (b) watching television (TV) while eating lunch or dinner; (c) eating while standing. These were grouped together based on conceptual similarity, reflecting simultaneous engagement in another activity during food consumption, rather than on statistical grouping procedures such as factor or cluster analysis [20]. However, no formal psychometric validation (e.g., factor analysis, internal consistency) of this composite was performed in the present sample, and eating while standing may capture situational or time-constrained eating rather than attentional distraction per se. Each item was rated using a four-point Likert scale ranging from 0 to 3, with higher scores indicating greater engagement in eating distractions. A composite “global eating distractions” was obtained by summing the three items, yielding a total score ranging from 0 to 9 points. 

#### 2.2.2. Chronotype

Chronotype was assessed using the Morningness–Eveningness Scale for Children (MESC), a Spanish adaptation of the original Morningness–Eveningness Questionnaire (MEQ). The instrument evaluates circadian preference through items assessing alertness and morning functioning in everyday situations (e.g., “How do you feel during the first 30 min after waking up?”), with response options organized on 4–5 point Likert-type scales ranging from strong disagreement to strong agreement. Total scores range from 10 to 42, with greater scores indicating morning preference and lower scores indicating evening type. Chronotype categories were defined using the following cut-offs: ≤20 (evening types), 21–28 points (intermediate types), and ≥29 points (morning types) [21]. Internal consistency of the MESC in the present sample was Cronbach’s α = 0.73, broadly consistent with previously reported values (e.g., α = 0.82) [22].

#### 2.2.3. Covariates

The following variables were included as covariates: age, sex, body mass index (BMI), physical activity, sedentary behavior, energy intake, socioeconomic status, and sleep patterns. Covariates were selected a priori based on their established associations with both chronotype and eating behaviors. Physical activity and sedentary behavior were included given the consistent evidence linking evening chronotype with lower physical activity and higher recreational screen time, which may independently increase exposure to television or device use during meals [23]. Sleep duration was included because shorter sleep is both a defining correlate of evening chronotype and an independent predictor of impaired self-regulation and attentional control, which may increase susceptibility to mealtime distraction [17,24]. Energy intake was adjusted for as a proxy for overall dietary pattern, given that distracted eating has been associated with altered satiety cues and intake volume [10]. Socioeconomic status was included as a potential confounder of both chronotype (via household routines and parental work schedules) and access to screens/devices at home [25]. BMI was included given its bidirectional associations with both chronotype and eating behaviors [9,14].

Participants self-reported their sex and age, while BMI was determined using standard anthropometric measurements of weight and height. BMI z scores were computed according to the World Health Organization criteria [26]. Age was included as a continuous covariate in all adjusted models; the median (Mdn) age reported in Table 1 is descriptive only and was not used to categorize participants for analysis. Socioeconomic status was assessed through the Family Affluence Scale (FAS-III), which evaluates household wealth on six criteria: number of bedrooms, vehicles, bathrooms, computers, vacations, and dishwashers, with a total score ranging from 0 to 13, where higher values indicate greater affluence [27]. Energy intake was estimated using a validated self-administered food frequency questionnaire (FFQ) designed for the Spanish adolescent population [28]. Physical activity and sedentary behavior were evaluated using the Spanish version of the Youth Activity Profile (YAP-S), a questionnaire that records activity patterns over seven days, classifying behaviors into school-related activities, extracurricular physical activities, and sedentary habits such as screen time [29]. Sleep duration was based on self-reported habitual bedtime and wake-up times on weekdays and weekends using the formula:

$$Sleep\;duration=\frac{\left(weekday\;sleep\;duration\times 5\right)+(weekend\;sleep\;duration\times 2)}{7}$$

### 2.3. Statistical Analysis

Statistical analyses were conducted using R statistical software (version 4.4.1; R Core Team in Vienna, Austria) and RStudio (2024.04.1 + 748; Posit in Boston, MA, USA). Continuous variables were expressed as Mdn and interquartile range (IQR), while categorical variables were expressed as numbers (n) and percentages (%). The distribution of variables was assessed using visual inspection of density and Q–Q plots, together with normality tests. Participant characteristics were described overall and stratified by chronotype category (evening, intermediate, and morning type); differences across chronotype categories were tested using Kruskal–Wallis tests for continuous variables and chi-square tests for categorical variables (Table 1). Interaction between chronotype and sex in relation to eating distraction scores was examined, and as no significant interaction was detected (*p* > 0.05), analyses were conducted combining boys and girls. Included and excluded participants were compared on available sociodemographic and lifestyle characteristics using Mann–Whitney U tests for continuous variables and chi-square tests for categorical variables (Table S5).

In this analysis, the chronotype category was the independent variable, and eating distractions (global score and individual items) were the dependent variables. Ordinal regression models (proportional odds) were used to examine associations between chronotype and eating distractions (global score and each item: eating while using a phone or social networks, watching TV during lunch or dinner, and eating while standing). Because the composite score was derived from a conceptual, non-statistically validated grouping of items (Section 2.2.1), each individual item was also modeled separately as a secondary outcome, to assess whether the association between chronotype and eating distractions was consistent across behaviors or driven by specific items. Odds ratios (ORs) with 95% confidence intervals (CIs) express the odds of being in a higher outcome category (e.g., higher frequency) for intermediate and morning type versus evening type (reference). Both unadjusted and multivariable-adjusted models were estimated. Adjusted models included sex, age, socioeconomic status (FAS-III), body mass index (BMI z-score, World Health Organization [WHO] criteria), physical activity, sedentary behavior, sleep duration, and energy intake. Because the outcome variables are ordered categories, ordinal regression was preferred over linear regression. The proportional odds assumption was checked with the Brant test and was tenable for the outcomes. Predicted probabilities from the fitted ordinal models were obtained to illustrate the association between chronotype and response category. As a sensitivity analysis, models were re-estimated using the continuous MESC score (higher scores indicating greater morningness) instead of chronotype categories, with the same covariate adjustment (Table S6).

## 3. Results

Table 1 presents the descriptive characteristics of the study participants according to chronotype status. A total of 820 Spanish youths (Mdn age: 14 years; IQR: 13.0–15.0) were included in the analysis, of whom 97 (11.8%) were classified as evening type, 401 (48.9%) as intermediate type and 322 (39.3%) as morning type. The overall chronotype (MESC) score had a Mdn of 26.0 points (IQR = 23.0–29.0). Several characteristics differed across chronotype categories: sex distribution (*p* < 0.001), physical activity (*p* < 0.001), sedentary behavior (*p* < 0.001), and sleep duration (*p* < 0.001). Evening-type adolescents showed lower physical activity, higher sedentary behavior, and shorter sleep duration than morning-type adolescents. Age, family affluence, energy intake, and BMI z-score did not differ significantly by chronotype (all *p* > 0.05). Eating distraction scores also differed across chronotype categories (global score, *p* < 0.001), with evening-type adolescents showing the least favorable distribution (Mdn = 3.0; IQR = 3.0–4.0) compared with morning-type adolescents (Mdn = 3.0; IQR = 2.0–3.0). Phone/social network use during meals (*p* < 0.001) and television viewing during meals (*p* = 0.002) differed by chronotype, whereas eating while standing did not (*p* > 0.9). Television viewing during meals was the most frequently reported eating distraction overall (Mdn = 2.0; IQR = 1.0–3.0). Compared with excluded adolescents, those included in the analytical sample were slightly younger, more often girls, and had modestly higher family affluence and longer sleep duration, whereas the MESC scores and chronotype distribution (among participants with available chronotype data) did not differ significantly (Table S5). In sensitivity analyses treating the MESC score as a continuous exposure, greater morningness was associated with lower odds of higher global eating distractions (OR = 0.95; 95% CI 0.92–0.98; *p* < 0.001), lower odds of phone/social network use during meals (OR = 0.96; 95% CI 0.93–0.99; *p* = 0.005), and lower odds of television viewing during meals (OR = 0.97; 95% CI 0.94–1.00; *p* = 0.022). No association was observed for eating while standing (Table S6).

**Figure 1:**
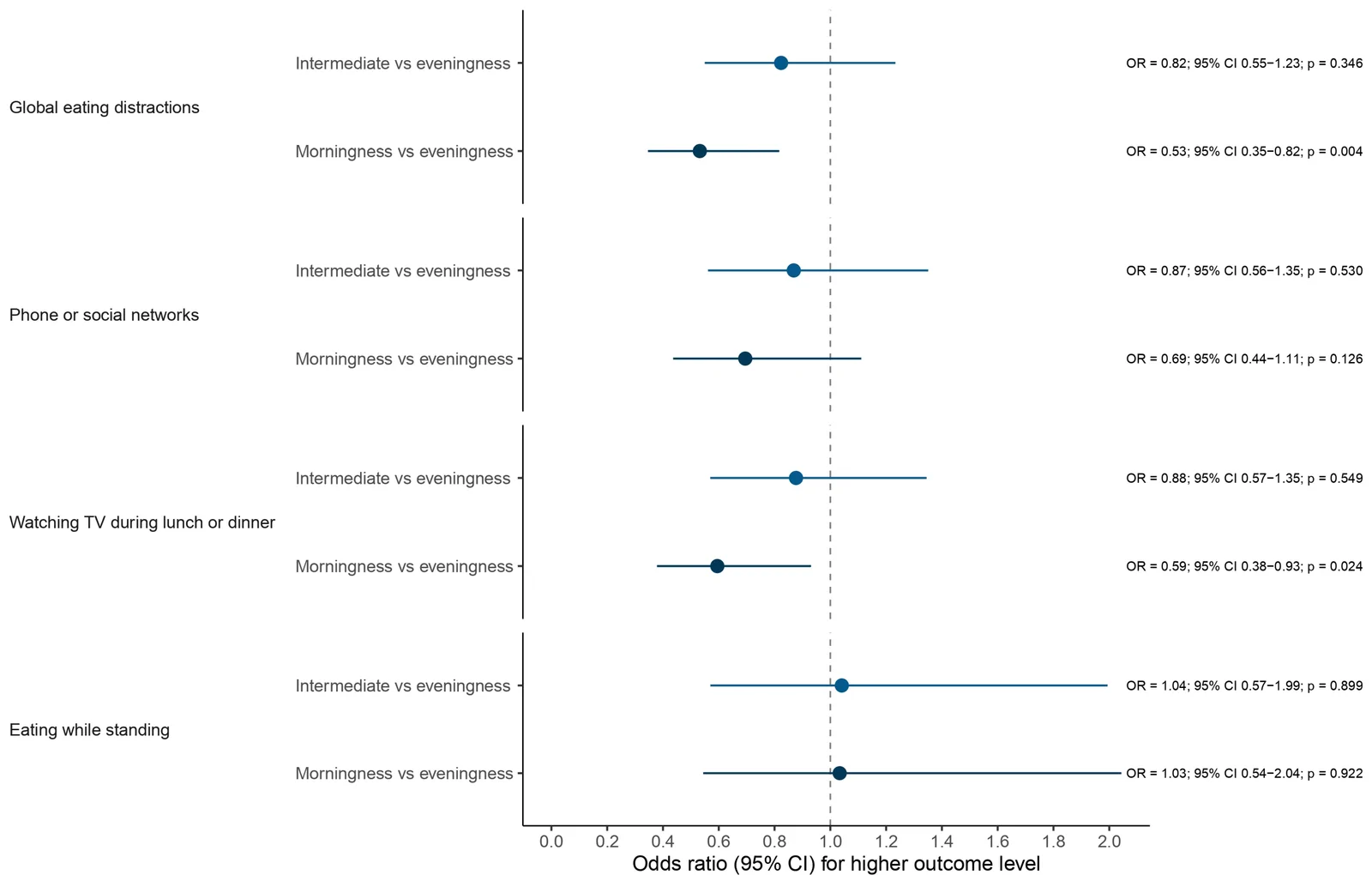
Association between global and individual eating distractions and chronotype status among adolescents (adjusted ordinal models). Adjusted for sex, age, socioeconomic status, body mass index z-score, sleep duration, physical activity, sedentary behavior and energy intake. Unadjusted estimates are reported in Tables S1 and S2. CI, confidence interval; TV, television.

**Figure 2:**
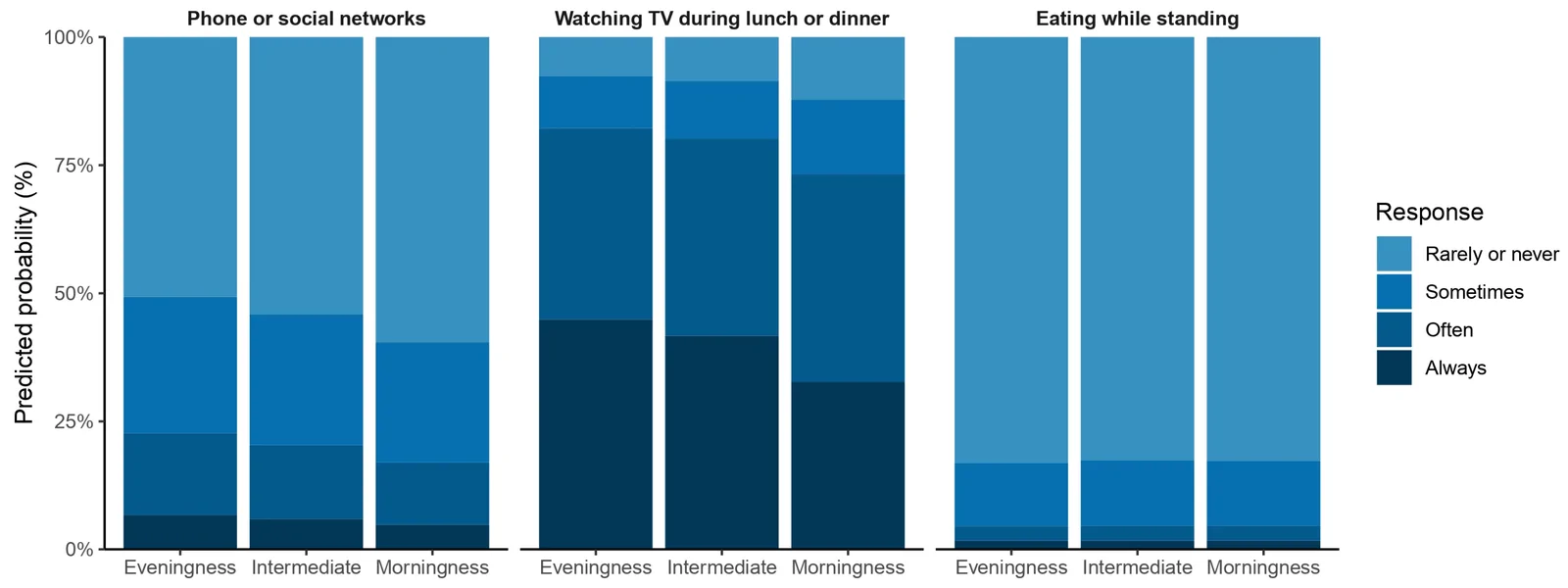
Predicted probabilities of response category by chronotype for each eating distraction item (ordinal regression, proportional odds). Adjusted for sex, age, socioeconomic status, body mass index z-score, sleep duration, physical activity, sedentary behavior and energy intake. TV, television.

Figure 1 displays the adjusted odds ratios from ordinal regression models, while Figure 2 presents the predicted probabilities for both the global score and individual eating distraction items according to chronotype status. In unadjusted models, morningness was associated with lower odds of a higher category for global eating distractions (OR = 0.41; 95% CI 0.27–0.61; *p* < 0.001) and for watching TV during meals (OR = 0.54; 95% CI 0.35–0.82; *p* = 0.004); phone/social network use during meals was also significant in the unadjusted model (OR = 0.47; 95% CI 0.31–0.72; *p* < 0.001). After multivariable adjustment, morningness remained associated with lower odds of a higher category for global eating distractions (OR = 0.53; 95% CI 0.35–0.82; *p* = 0.004) and for watching TV during meals (OR = 0.59; 95% CI 0.38–0.93; *p* = 0.024), whereas the association for phone/social networks was attenuated and no longer statistically significant (OR = 0.69; 95% CI 0.44–1.11; *p* = 0.126). No significant associations were found for eating while standing in either unadjusted or adjusted models. Intermediate chronotype was not significantly associated with any eating distraction outcome in adjusted models. Detailed unadjusted and adjusted results are presented in Tables S1 and S2 (ORs), Tables S3 and S4 (predicted probabilities), and Tables S5 and S6 (attrition comparison and continuous MESC sensitivity analysis).

## 4. Discussion

To our knowledge, this is the first study to examine the association between chronotype and eating distractions among Spanish adolescents. Evening-type adolescents showed higher eating distraction scores than morning-type adolescents. This association appeared to be primarily driven by television viewing during meals, for which morning-type adolescents reported significantly lower scores. These findings highlight chronotype as a potentially relevant dimension when examining contextual eating behaviors in adolescents. Given the cross-sectional design of this study, causal inference cannot be established. The observed associations may reflect chronotype influencing mealtime behaviors, reverse causation, for instance, established eating or screen-use habits shaping sleep–wake timing, or shared underlying factors (e.g., family routines, parental supervision, socioeconomic context) that were not fully captured in this analysis.

Direct evidence examining the relationship between chronotype and eating distractions in adolescents remains scarce. Previous studies have primarily explored chronotype in relation to broader eating behaviors, with evening-type individuals showing greater emotional eating, disinhibition, and obesity risk, albeit without specifically addressing mealtime distractions [30]. Other research has focused on the impact of screen use during meals, linking it to poorer dietary quality, higher intake of sugar-sweetened beverages, and unhealthy eating patterns [4,31,32]. While these findings provide indirect support for the relevance of mealtime distractions, they do not consider circadian preference as a potential modifying factor. In this context, our results extend previous research by suggesting that evening-type adolescents may be particularly prone to television viewing during meals, whereas other distraction behaviors did not differ across chronotype categories.

Television viewing during meals emerged as the primary eating distraction associated with chronotype in our study, with evening-type adolescents showing higher scores than their morning-type peers. This finding is consistent with previous research indicating that evening chronotypes are more prone to disordered eating behaviors and poorer diet quality [33]. Evidence from a Chinese population further supports this pattern, showing that evening-type individuals are less likely to consume fruits and vegetables, more likely to skip or have smaller breakfasts, and more likely to watch television during meals, behaviors linked to increased childhood obesity risk [34]. Moreover, television use during meals has been associated with contextual factors such as parental education level, suggesting that environmental influences may also interact with chronotype-related behaviors [34]. These findings suggest that evening-type adolescents may be particularly vulnerable to unhealthy mealtime habits, with television viewing during meals potentially contributing to poor dietary quality.

Notably, only television viewing during meals was significantly associated with chronotype, while mobile device use and eating while standing were not. This may reflect that television viewing during meals is a more habitual, household-level behavior closely tied to evening routines typical of later chronotypes, whereas smartphone use during meals may be more uniformly prevalent across chronotype groups given its near-ubiquitous use among adolescents [4,32]. Eating while standing, in contrast, may be driven more by time pressure or schedule constraints than by circadian preference. These findings suggest that eating distractions are not a homogeneous behavioral category and that chronotype-related interventions may need to target specific mealtime behaviors rather than distraction broadly. Similarly, the observed association between evening chronotype and television viewing during meals may be partially explained by circadian-related differences in alertness, behavioral regulation, and daily routines [24]. Evening-type adolescents often exhibit later sleep timing and greater social jetlag, patterns that have been linked to irregular meal schedules, less structured eating behaviors and metabolic disturbances [14]. Such circadian misalignment may increase the likelihood of engaging in screen-based activities during meals, particularly in the evening hours. Furthermore, evening meals may coincide with periods of higher arousal in evening chronotypes, potentially favoring multitasking behaviors and reduced focus on eating itself. Previous evidence indicates that greater circadian alignment is associated with healthier and more structured eating behaviors, whereas circadian misalignment is linked to less favorable eating patterns [35,36]. Although the underlying mechanisms remain to be clarified, these findings suggest that biological timing and lifestyle factors may be linked with mealtime behaviors during adolescence.

Sedentary behavior and sleep duration were both significantly associated with eating distractions in adjusted models. These variables may act as confounders of the chronotype–eating distraction association or, alternatively, as mediators on the causal pathway from chronotype to eating distractions, given their established bidirectional relationship with circadian preference [33,35]. The cross-sectional design does not allow these possibilities to be distinguished, and this should be considered when interpreting the adjusted estimates.

These findings may be interpreted in light of circadian and behavioral mechanisms. Evening-type adolescents may spend more time in the evening using screens (e.g., television or mobile devices) and may therefore be more likely to combine meals with these activities. Social jetlag and delayed sleep timing could also be associated with less structured mealtimes and more frequent eating in front of screens. The lack of association observed for eating while standing may suggest that this item reflects a different construct (e.g., eating context) rather than distraction per se, or that chronotype may be less relevant for this behavior. Future research should examine these potential mechanisms and further validate the eating distraction score in adolescent populations.

### Limitations and Strengths

Despite some limitations, this study has several strengths. These include the use of a well-characterized adolescent cohort from the EHDLA study, the application of validated instruments to assess behavioral variables, and the use of ordinal regression (proportional odds), which is appropriate for ordered outcome categories, enhancing the validity and reliability of the findings. Several limitations should also be considered when interpreting these results. First, the cross-sectional design does not allow causal inferences or determination of the directionality of the observed associations. Longitudinal studies are needed to clarify whether an evening chronotype predisposes adolescents to engage in eating distraction behaviors or whether these behaviors influence circadian preference. Second, all exposure, outcome, and covariate data were self-reported, introducing potential recall bias and social desirability bias, particularly relevant for dietary intake and screen-based behaviors, which adolescents may underreport. Future studies incorporating objective measures (e.g., accelerometry for sedentary behavior, actigraphy for sleep, or observational mealtime recordings) would strengthen these findings. Third, the composite eating distraction score assumes these three behaviors reflect a single underlying construct; the finding that only television viewing was significantly associated with chronotype suggests this assumption should be interpreted with caution, and future studies should assess the psychometric structure of this measure. Fourth, because sedentary behavior may be conceptually and temporally related to eating distractions (e.g., television viewing during meals is itself a sedentary behavior), its inclusion as a covariate may have resulted in some degree of overadjustment. Fifth, pubertal/sexual maturation status was not assessed in this study. Given known differences in sleep timing, energy requirements, and body composition across pubertal stages, residual confounding by maturation status cannot be excluded, independent of chronological age. Sixth, dietary intake was assessed via FFQ, which is subject to misreporting. However, evidence on the correlates of underreporting in adolescents is mixed and appears to depend on the reference method used to validate self-report. For instance, studies using FFQ- or recall-based indices (e.g., energy intake relative to estimated basal metabolic rate) have reported greater underreporting among girls than boys [37], whereas studies using doubly labeled water (an objective reference standard) have reported the opposite pattern, with greater underreporting among boys [38]. Other large studies using 24 h recalls or multi-day diet records have found no significant sex difference in underreporting [39]. In addition, of the initial 1378 participants, 558 (40.5%) were excluded because of incomplete data; included and excluded adolescents differed in age, sex, socioeconomic status, and sleep duration, so selection bias cannot be ruled out, although chronotype scores did not differ among those with available MESC data (Table S5). Furthermore, neurodevelopmental and psychiatric conditions (including ADHD, autism spectrum disorder, clinically diagnosed depression or anxiety, eating disorders, and sleep disorders) were not systematically recorded and therefore could not be accounted for, leaving residual confounding possible. Finally, although analyses were adjusted for multiple relevant covariates, residual confounding from unmeasured factors (e.g., psychological characteristics, family environment, or other lifestyle behaviors) cannot be ruled out.

## 5. Conclusions

This study suggests that adolescents with an evening chronotype may be more likely to engage in mealtime distractions, particularly television viewing, compared with their morningness and intermediate peers. These findings highlight the potential relevance of circadian preference in shaping contextual eating behaviors during adolescence. Given the cross-sectional design and modest effect sizes observed, these findings should be interpreted as hypothesis-generating. Longitudinal studies are needed before chronotype-based screening is incorporated into mealtime behavior interventions.

## Figures and Tables

**Table 1:** Descriptive data of the study participants according to chronotype status.

Variable	Overall N = 820	Eveningness N = 97	Intermediate N = 401	Morningness N = 322	*p*-Value
Age (years)	14.0 (13.0, 15.0)	14.0 (13.0, 15.0)	14.0 (13.0, 15.0)	14.0 (13.0, 15.0)	0.065
Sex					<0.001
Boys	365 (45%)	28 (29%)	172 (43%)	165 (51%)	
Girls	455 (55%)	69 (71%)	229 (57%)	157 (49%)	
FAS-III (score)	8.0 (7.0, 10.0)	9.0 (7.0, 10.0)	8.0 (7.0, 10.0)	8.0 (7.0, 10.0)	0.260
YAP-S physical activity (score)	2.6 (2.2, 3.0)	2.3 (1.8, 2.8)	2.6 (2.1, 3.0)	2.7 (2.3, 3.2)	<0.001
YAP-S sedentary behaviors (score)	2.6 (2.2, 3.0)	2.8 (2.4, 3.4)	2.6 (2.2, 3.0)	2.4 (2.0, 2.8)	<0.001
Overall sleep duration (minutes)	497.1 (458.6, 527.1)	467.1 (428.6, 518.6)	497.1 (454.3, 527.1)	505.7 (471.4, 535.7)	<0.001
Energy intake (kcal)	2581.9 (1954.6, 3442.5)	2867.9 (1970.7, 3713.0)	2593.0 (1967.3, 3400.8)	2477.9 (1887.9, 3400.9)	0.145
BMI (z-score)	0.9 (–0.03, 1.9)	0.9 (–0.01, 1.9)	0.9 (-0.1, 1.8)	0.9 (0.0, 1.9)	0.572
Eating while using a phone or social networks (score)	0.0 (0.0, 1.0)	1.0 (0.0, 2.0)	0.0 (0.0, 1.0)	0.0 (0.0, 1.0)	<0.001
Eating while watching TV during lunch or dinner (score)	2.0 (1.0, 3.0)	2.0 (2.0, 3.0)	2.0 (2.0, 3.0)	2.0 (1.0, 3.0)	0.002
Eating while standing (score)	0.0 (0.0, 0.0)	0.0 (0.0, 0.0)	0.0 (0.0, 0.0)	0.0 (0.0, 0.0)	0.993
Eating distractions (score)	3.0 (2.0, 4.0)	3.0 (3.0, 4.0)	3.0 (2.0, 4.0)	3.0 (2.0, 3.0)	<0.001

Median (interquartile range) or numbers (percentage). *p* values from Kruskal–Wallis tests (continuous variables) or chi-square tests (categorical variables). Body mass index z-scores were computed according to the World Health Organization criteria [26]. BMI, body mass index; FAS-III, Family Affluence Scale-III; YAP-S, Spanish Youth Activity Profile.

## Data Availability

The data utilized in this study can be accessed upon reasonable request from the corresponding author. Since the data involves minors, privacy and confidentiality must be maintained.

## Supplementary Materials

The following supporting information can be downloaded at: https://www.mdpi.com/article/10.3390/nu18172763/s1, Table S1. Unadjusted and adjusted proportional odds models of the association between chronotype status and global eating distractions among adolescents. Table S2. Unadjusted and adjusted proportional odds models of the association between chronotype status and eating distractions by item (phone/social networks, watching TV during lunch or dinner, eating while standing). Table S3. Predicted probabilities of global eating distractions score (0–9 points) by chronotype (ordinal regression, proportional odds). Adjusted for sex, age, socioeconomic status, body mass index z-score, sleep duration, physical activity, sedentary behavior and energy intake. Table S4. Predicted probabilities of response category by chronotype for each eating distraction item (ordinal regression, proportional odds). Table S5. Comparison of included and excluded participants. Table S6. Sensitivity analysis using continuous MESC score.

## Abbreviations

The following abbreviations are used in this manuscript:

EHDLA	Eating Healthy and Daily Life Activities
OR	Odds Ratio
CI	Confidence Interval
TV	Television
MESC	Morningness–Eveningness Scale for Children
MEQ	Morningness–Eveningness Questionnaire
BMI	Body Mass Index
WHO	World Health Organization
FAS-III	Family Affluence Scale III
FFQ	Food Frequency Questionnaire
YAP-S	Youth Activity Profile—Spanish version
IQR	Interquartile Range
Mdn	Median
ID	Identification Number (código de aprobación ética)
