# Supplementary material for: Is Chronotype Related to Eating Distractions Among Adolescents? The EHDLA Study
Source: Nutrients. 2026 Aug 24;18(17):2763. doi: 10.3390/nu18172763 (PMC13567580; doi:10.3390/nu18172763)
Supplement: Supplementary file 1 [file nutrients-18-02763-s001.zip › nutrients-4396731-supplementary.pdf]

Supplementary Material

Table S1. Unadjusted and adjusted proportional odds models of the association between chronotype status and global eating distractions among adolescents.

Chronotype	Unadjusted OR (95% CI)	p (unadjusted)	Adjusted OR (95% CI)	p (adjusted)
Eveningness	Ref.		Ref.	
Intermediate	0.68 (0.46-1.01)	0.054	0.82 (0.55-1.23)	0.346
Morningness	0.41 (0.27-0.61)	<0.001	0.53 (0.35-0.82)	0.004

Reference: eveningness. Unadjusted and adjusted ORs are shown. Adjusted models controlled for sex, age, socioeconomic status (FAS-III), BMI z-score (WHO), sleep duration, physical activity, sedentary behavior, and energy intake. BMI, body mass index; CI, confidence interval; FAS-III, Family Affluence Scale-III; OR, odds ratio; Ref., reference; WHO, World Health Organization; YAP-S, Spanish Youth Active Profile.

Table S2. Unadjusted and adjusted proportional odds models of the association between chronotype status and eating distractions by item (phone/social networks, watching TV during lunch or dinner, eating while standing).

Outcome	Chronotype	Unadjusted OR (95% CI)	p (unadjusted)	Adjusted OR (95% CI)	p (adjusted)
	Eveningness	Ref.		Ref.	
Eating while using a phone or social networks	Intermediate	0.68 (0.45-1.04)	0.072	0.87 (0.56-1.35)	0.530
	Morningness	0.47 (0.31-0.72)	<0.001	0.69 (0.44-1.11)	0.126
	Eveningness	Ref.		Ref.	
Eating while watching TV during lunch or dinner	Intermediate	0.81 (0.54-1.23)	0.325	0.88 (0.57-1.35)	0.549
	Morningness	0.54 (0.35-0.82)	0.004	0.59 (0.38-0.93)	0.024
	Eveningness	Ref.		Ref.	
Eating while standing	Intermediate	1.03 (0.58-1.92)	0.916	1.04 (0.57-1.99)	0.899
	Morningness	1.01 (0.56-1.92)	0.962	1.03 (0.54-2.04)	0.922

Reference: eveningness. Unadjusted and adjusted ORs are shown. Adjusted models controlled for sex, age, socioeconomic status (FAS-III), BMI z-score (WHO), sleep duration, physical activity, sedentary behavior, and energy intake. BMI, body mass index; CI, confidence interval; FAS-III, Family Affluence Scale-III; OR, odds ratio; WHO, World Health Organization; YAP-S, Spanish Youth Active Profile.

Table S3. Predicted probabilities of global eating distractions score (0–9 points) by chronotype (ordinal regression, proportional odds). Adjusted for sex, age, socioeconomic status, body mass index z-score, sleep duration, physical activity, sedentary behavior and energy intake.

**Chronotype**	**0 points % (95% CI)**	**1 point % (95% CI)**	**2 points % (95% CI)**	**3 points % (95% CI)**	**4 points % (95% CI)**	**5 points % (95% CI)**	**6 points % (95% CI)**	**7 points % (95% CI)**	**8 points % (95% CI)**	**9 points % (95% CI)**
Eveningness	4.3 (2.6-6.5)	5.5 (3.7-7.7)	19.4 (15.2-24.8)	30.9 (27.2-34.3)	19.8 (16.1-23.7)	12.3 (8.5-16.0)	5.7 (3.3-8.8)	0.9 (0.2-1.7)	0.8 (0.2-1.6)	0.5 (0.0-1.1)
Intermediate	5.2 (3.6-7.0)	6.5 (4.8-8.5)	21.7 (18.4-25.6)	31.2 (28.1-34.6)	18.2 (14.8-21.8)	10.6 (8.1-13.0)	4.8 (3.1-6.4)	0.8 (0.2-1.3)	0.6 (0.2-1.2)	0.4 (0.0-0.9)
Morningness	7.8 (5.5-10.6)	9.2 (6.9-11.7)	26.7 (23.1-30.9)	30.2 (27.1-33.7)	14.3 (11.4-17.2)	7.5 (5.5-9.5)	3.2 (1.9-4.4)	0.5 (0.1-0.9)	0.4 (0.1-0.8)	0.2 (0.0-0.6)

%, percentage; CI, confidence interval; TV, television. Adjusted for sex, age, socioeconomic status, body mass index z-score, sleep duration, physical activity, sedentary behavior and energy intake.

Table S4. Predicted probabilities of response category by chronotype for each eating distraction item (ordinal regression, proportional odds).

Outcome	Chronotype	Rarely or never % (95% CI)	Sometimes % (95% CI)	Often % (95% CI)	Always % (95% CI)
Phone or social networks	Eveningness	50.6 (39.5-62.2)	26.6 (22.1-30.7)	16.0 (10.9-21.9)	6.7 (3.9-10.8)
Phone or social networks	Intermediate	54.2 (48.1-60.5)	25.5 (22.5-28.8)	14.4 (11.5-17.9)	5.9 (4.1-7.8)
Phone or social networks	Morningness	59.6 (52.6-65.7)	23.4 (20.0-27.6)	12.2 (9.4-15.1)	4.8 (3.1-6.8)
Watching TV during lunch or dinner	Eveningness	7.6 (4.7-11.4)	10.1 (6.6-14.1)	37.2 (31.4-42.2)	45.0 (34.5-55.9)
Watching TV during lunch or dinner	Intermediate	8.6 (6.1-10.9)	11.2 (8.6-13.4)	38.5 (35.3-42.3)	41.8 (36.6-47.5)
Watching TV during lunch or dinner	Morningness	12.1 (8.9-15.2)	14.5 (11.2-17.6)	40.6 (37.3-44.6)	32.8 (27.1-38.6)
Eating while standing	Eveningness	83.2 (73.1-91.4)	12.4 (6.7-19.2)	2.8 (1.2-5.4)	1.7 (0.6-3.4)
Eating while standing	Intermediate	82.5 (77.8-87.7)	12.9 (9.0-16.5)	2.9 (1.6-4.8)	1.7 (0.8-2.7)
Eating while standing	Morningness	82.6 (77.1-87.4)	12.8 (9.3-16.7)	2.9 (1.6-4.4)	1.7 (0.8-3.1)

%, predictive probability; CI, confidence interval; TV, television. Adjusted for sex, age, socioeconomic status, body mass index z-score, sleep duration, physical activity, sedentary behavior and energy intake.

Table S5. Sociodemographic and lifestyle characteristics of adolescents included in the analytical sample (n = 820) versus those excluded due to incomplete data (n = 558).

**Variable**	**Included**	**n**	**Excluded**	**n**	**p**
Age (years)	14.0 (13.0-15.0)	820	15.0 (13.0-17.0)	558	<0.001
FAS-III (score)	8.0 (7.0-10.0)	820	8.0 (6.0-9.0)	291	0.031
Sleep duration (min)	497.1 (458.6-527.1)	820	484.3 (441.4-522.9)	281	0.003
YAP-S physical activity (score)	2.6 (2.2-3.0)	820	2.7 (2.1-3.1)	262	0.708
YAP-S sedentary behaviors (score)	2.6 (2.2-3.0)	820	2.6 (2.2-3.0)	262	0.059
Energy intake (kcal)	2581.9 (1954.7-3442.0)	820	2754.4 (2025.9-3972.0)	116	0.132
BMI (kg/m^2)	21.7 (19.3-25.3)	820	21.8 (19.4-25.6)	441	0.909
MESC score	26.0 (23.0-29.0)	820	26.0 (23.0-28.0)	80	0.140
Sex, boys n (%)	365 (44.5%)	820	315 (56.5%)	558	<0.001
Sex, girls n (%)	455 (55.5%)	820	243 (43.5%)	558	
Chronotype, Eveningness n (%) *	97 (11.8%)	820	13 (16.2%)	80	0.348
Chronotype, Intermediate n (%) *	401 (48.9%)	820	41 (51.2%)	80	
Chronotype, Morningness n (%) *	322 (39.3%)	820	26 (32.5%)	80	

Values for continuous variables are median (interquartile range). P values from Mann–Whitney U tests (continuous) or chi-square tests (categorical). * Chronotype categories were compared among participants with available Morningness–Eveningness category data (included n = 820; excluded n = 80). BMI, body mass index; FAS-III, Family Affluence Scale-III; MESC, Morningness–Eveningness Scale for Children; YAP-S, Youth Activity Profile–Spain.

Table S6. Sensitivity analysis: proportional odds models using the continuous MESC score (per one-point increase; higher scores indicate greater morningness) and eating distractions.

Outcome	OR (95% CI)	p-value	n
Global eating distractions	0.95 (0.92-0.98)	<0.001	820
Phone or social networks	0.96 (0.93-0.99)	0.005	820
Watching TV during lunch or dinner	0.97 (0.94-1.00)	0.022	820
Eating while standing	1.01 (0.96-1.05)	0.776	820

CI, confidence interval; MESC, Morningness–Eveningness Scale for Children; OR, odds ratio; TV, television. Models adjusted for sex, age, socioeconomic status (FAS-III), body mass index z-score (WHO), sleep duration, physical activity, sedentary behavior, and energy intake. OR < 1 indicates lower odds of being in a higher eating-distraction category per one-point increase in MESC (i.e., greater morningness).
